# Comparative diversity of microbiomes and Resistomes in beef feedlots, downstream environments and urban sewage influent

**DOI:** 10.1186/s12866-019-1548-x

**Published:** 2019-08-27

**Authors:** Rahat Zaheer, Steven M. Lakin, Rodrigo Ortega Polo, Shaun R. Cook, Francis J. Larney, Paul S. Morley, Calvin W. Booker, Sherry J. Hannon, Gary Van Domselaar, Ron R. Read, Tim A. McAllister

**Affiliations:** 1 Agriculture and Agri-Food Canada, Lethbridge Research and Development Centre, 5403 1 Ave, Lethbridge, AB T1J 4P4 Canada; 2 Department of Clinical Sciences, Colorado State University, Fort Collins, CO 80523 USA; 3 Alberta Agriculture and Forestry, 100, 5401 – 1st Avenue South, Lethbridge, AB T1J 4V6 Canada; 4 Feedlot Health Management Services, Okotoks, AB Canada; 5 National Microbiology Laboratory, Public Health Agency of Canada, 1015 Arlington Street, Winnipeg, MB R3E 3R2 Canada; 6 Cumming School of Medicine, University of Calgary, 3280 Hospital Drive NW, Calgary, AB T2N 4N1 Canada

**Keywords:** Microbiome, Antimicrobial resistance, Metal and biocide resistance, Cattle production, Wastewater

## Abstract

**Background:**

Comparative knowledge of microbiomes and resistomes across environmental interfaces between animal production systems and urban settings is lacking. In this study, we executed a comparative analysis of the microbiota and resistomes of metagenomes from cattle feces, catch basin water, manured agricultural soil and urban sewage.

**Results:**

Metagenomic DNA from composite fecal samples (FC; *n* = 12) collected from penned cattle at four feedlots in Alberta, Canada, along with water from adjacent catchment basins (CB; *n* = 13), soil (*n* = 4) from fields in the vicinity of one of the feedlots and urban sewage influent (SI; *n* = 6) from two municipalities were subjected to Illumina HiSeq2000 sequencing. Firmicutes exhibited the highest prevalence (40%) in FC, whereas Proteobacteria were most abundant in CB (64%), soil (60%) and SI (83%). Among sample types, SI had the highest diversity of antimicrobial resistance (AMR), and metal and biocide resistance (MBR) classes (13 & 15) followed by FC (10 & 8), CB (8 & 4), and soil (6 & 1). The highest antimicrobial resistant (AMR) gene (ARG) abundance was harboured by FC, whereas soil samples had a very small, but unique resistome which did not overlap with FC & CB resistomes. In the beef production system, tetracycline resistance predominated followed by macrolide resistance. The SI resistome harboured β-lactam, macrolide, tetracycline, aminoglycoside, fluoroquinolone and fosfomycin resistance determinants. Metal and biocide resistance accounted for 26% of the SI resistome with a predominance of mercury resistance.

**Conclusions:**

This study demonstrates an increasing divergence in the nature of the microbiome and resistome as the distance from the feedlot increases. Consistent with antimicrobial use, tetracycline and macrolide resistance genes were predominant in the beef production system. One of the feedlots contributed both conventional (raised with antibiotics) and natural (raised without antibiotics) pens samples. Although natural pen samples exhibited a microbiota composition that was similar to samples from conventional pens, their resistome was less complex. Similarly, the SI resistome was indicative of drug classes used in humans and the greater abundance of mercury resistance may be associated with contamination of municipal water with household and industrial products.

**Electronic supplementary material:**

The online version of this article (10.1186/s12866-019-1548-x) contains supplementary material, which is available to authorized users.

## Background

Antimicrobials have played an important role in controlling bacterial infectious diseases in both humans and animals. In livestock, antimicrobials are used mainly for the treatment and prevention of disease as label claims for their use at sub-therapeutic levels to promote growth are being removed [1]. The worldwide consumption of antimicrobials in food animal production has been reported at ≥57 million kg with a projected increase to ≥95 million kilogram by 2030 [2]. In North American beef feedlots, a number of antimicrobials are administered to cattle, with macrolides and tetracyclines accounting for the majority of antimicrobial use (AMU) [3]. Bacteria residing in the bovine gastrointestinal tract may become resistant to these antibiotics and, once released into the environment, they may transfer antimicrobial resistance (AMR) genes (ARGs) to other bacteria including potential human pathogens [4, 5]. Furthermore, residual antibiotics may enter the environment through runoff from manure, where they may select for antimicrobial resistant bacteria [6, 7]. Consequently, it is not surprising that for almost every livestock-associated bacterial pathogen, resistance to at least one antimicrobial from each antimicrobial class has been reported [8].

Antimicrobials are not fully metabolized when administered to either humans or livestock. Gao et al. [9] estimated that up to 90% of many of the antibiotics used in livestock are excreted in urine or feces. Sewage treatment plants (STP) receive waste streams that contain a mixture of nutrients, metals, antibiotics, and industrial/household chemicals from a variety of sources [10]. Antimicrobials, antimicrobial resistant bacteria (ARB) and ARGs are frequently detected in STP [11, 12] and as a result these facilities have been identified as a potential hotspot for antibiotic resistance, where ARGs spread among bacteria via horizontal gene transfer. These biological pollutants are also released into the environment in STP effluent [13–15].

Knowledge of the microbiome and resistome within and between the environmental interface between animal production systems and urban centres is lacking. Information gained from an understanding of this interface could help support more prudent use of antimicrobials in livestock, more specifically, in defining targeted treatment options and distinguishing between essential and non-essential AMU to ensure safer food production practices.

Culture independent techniques, such as next generation sequencing (NGS) can be used to quantitatively assess the microbiota composition and its associated resistome. Advances in high-throughput NGS technologies have enabled rapid understanding of overall microbial ecology as well as occurrence and diversity of ARGs from diverse environments. Whole-metagenome shotgun analyses are accomplished by unrestricted sequencing of the genomes of most microorganisms present in a sample, including currently uncultured organisms. The present study describes the microbial metagenomes and resistomes of a variety of environmental samples from beef production to human-associated wastes (urban sewage). We utilize a NGS approach to inform surveillance as well as to improve the current understanding of the microbial community structure, the prevalence of ARGs within these microbial communities and to investigate overlaps between various components of the environmental spectrum.

## Results and discussion

All 35 samples (FC = 12, CB = 13, soil = 4 and SI = 6) were sequenced to an average of ~ 54 million reads per sample. This sequencing depth was found appropriate, as indicated by the saturation of novel taxa and ARGs in our previous study which investigated the microbiota and resistome of bovine fecal samples [16]. The average read quality score for samples in the present study ranged from 33 to 37, indicative of high quality reads. Of the total number of reads generated, 94–97% survived quality filtering and trimming across all datasets.

### Each sampling group exhibited distinct composition of microbiota

**Fig. 1:**
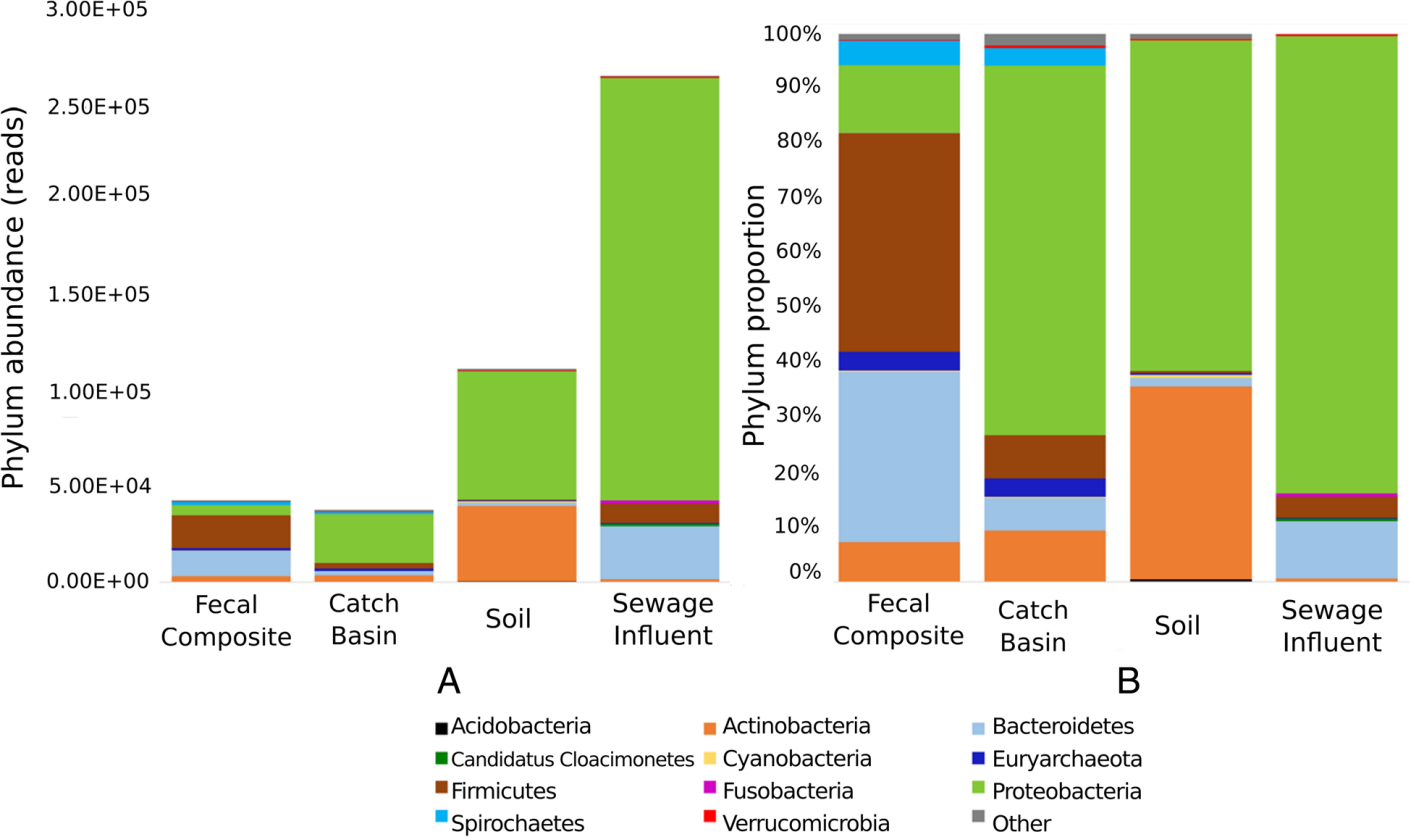
Abundance and relative proportion of microbial phyla in sample types. Abundance (**a**) is a measure of read counts aligning to various phyla (indicated by different colors) and normalized across samples whereas proportion (**b**) indicates percentage of each phylum in a sample type. The category ‘Other’ includes the rest of the low abundance phyla for each sample type

Across all samples 5.9% of total reads aligned to bacterial and archaeal species, representing 816 genera and 35 phyla. The proportion of prokaryote-associated (bacteria and archaea) raw (trimmed and quality filtered) reads arising from the total metagenomic raw reads varied among various sample types. Sewage influent (SI) had the highest number of prokaryote-associated reads, followed by soil, catch-basin (CB) water, and bovine feces (FC). For SI, 24.5% of the sequence reads were associated with bacteria and archaea, whereas soil, CB and FC had a much smaller proportion of prokaryote-associated reads (3.4, 4.5 and 2.1%, respectively), as revealed by the taxonomic classification via Kraken. The majority of remaining read fractions in these samples were uncharacterized, most likely originating from uncharacterized prokaryotes as well as eukaryotic organisms including algae, plants, small eukaryotes, avian or mammalian sources that are absent from the Kraken database. The comparatively high proportion of prokaryote-associated reads in SI is reflective of the very high density (2–10 g dry weight/L) of microorganisms within sewage [17]. Comparison of normalized data across all samples also supported the largest abundance of microbial taxa reads in SI, being 6.2, 6.7, and 2.4 fold higher than in FC, CB and soil, respectively (Fig. 1).

**Fig. 2:**
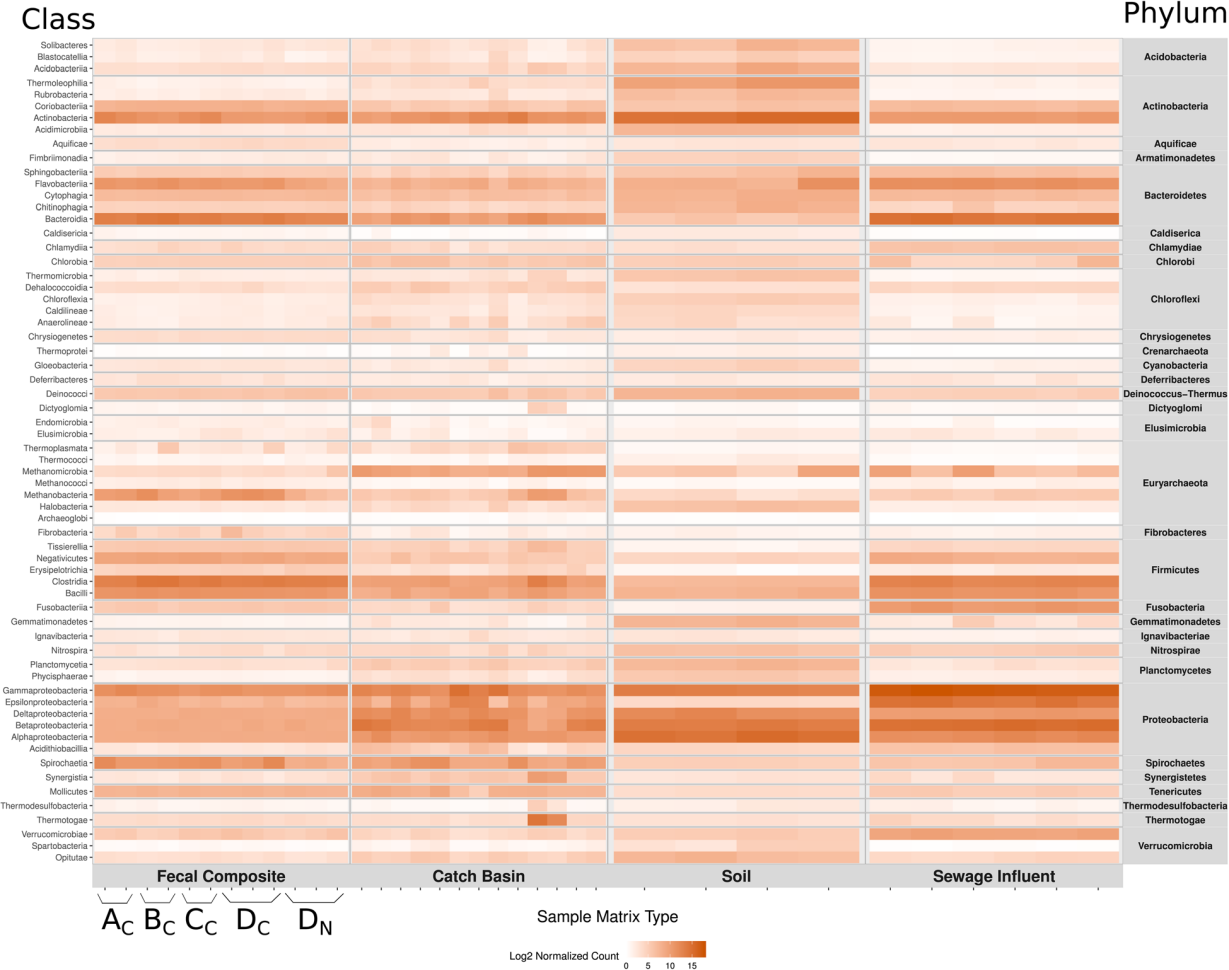
Heat map of prevalent taxonomic classes across all samples grouped by phyla. As described in methods section, fecal composite (FC) samples were obtained from four feedlots **a**, **b**, **c** and **d**. The subscript letters _C_ and _N_ denote conventional and natural practices, respectively

In FC, Firmicutes were the most abundant (39%) followed by Bacteroidetes (32%), Proteobacteria (11%), Actinobacteria (7%), Spirochaetes (5%) and Euryarchaeota (4%). These six phyla constituted 98.5% of the FC microbial community with Firmicutes and Bacteroidetes accounting for over 70% of the community. Predominance of Firmicutes and Bacteroidetes in livestock gastrointestinal tract microbiota is in agreement with other studies [18–21]. The most abundant classes of Firmicutes and Bacteroidetes in FC included Clostridia and Bacteroidia, respectively (Fig. 2) corresponding to 59% of prokaryotic reads, whereas Bacteroidaceae, Prevotellaceae, Methanobacteriaceae, Flavobacteriaceae, Clostridiaceae, Enterobacteriaceae were among the most abundant families (relative abundance range 12.6–7.5%). The five most predominant bacterial genera included *Prevotella*, *Bacteroides*, *Treponema*, *Bifidobacterium* and *Clostridium* (Table 1). *Methanobrevibacter* was the most prevalent genus from the archaeal phylum Euryarchaeota (Table 1). This genus has been previously characterized as hydrogenotrophic rumen methanogens [22]. *Methanobrevibacter* accounts for 80–85% of all methanobacterial reads in the cattle fecal methanogenic community [21, 23]; it is also the dominant methanogen in the rumen [24, 25].

**Table 1:** Top twenty most abundant genera identified for each sample type

	Fecal Composite			Catch Basin			Soil			Sewage influent		
No.	Genus	Phylum	Proportion	Genus	Phylum	Proportion	Genus	Phylum	Proportion	Genus	Phylum	Proportion
1	Prevotella	Bacteroidetes	12.02%	Thauera	Proteobacteria	12.20%	Bradyrhizobium	Proteobacteria	8.19%	Acinetobacter	Proteobacteria	29.16%
2	Bacteroides	Bacteroidetes	11.32%	Psychrobacter	Proteobacteria	9.83%	Streptomyces	Actinobacteria	5.47%	Aeromonas	Proteobacteria	16.25%
3	Treponema	Spirochaetes	6.76%	Corynebacterium	Actinobacteria	5.09%	Actinoplanes	Actinobacteria	4.60%	Arcobacter	Proteobacteria	8.68%
4	Bifidobacterium	Actinobacteria	5.67%	Arcobacter	Proteobacteria	4.38%	Mycobacterium	Actinobacteria	3.22%	Bacteroides	Bacteroidetes	8.13%
5	Methanobrevibacter	Euryarchaeota	5.08%	Sphaerochaeta	Spirochaetes	4.16%	Pseudomonas	Proteobacteria	3.15%	Moraxella	Proteobacteria	6.38%
6	Clostridium	Firmicutes	4.74%	Pseudomonas	Proteobacteria	3.73%	Rhodopseudomonas	Proteobacteria	2.69%	Acidovorax	Proteobacteria	4.75%
7	Chryseobacterium	Proteobacteria	4.31%	Fermentimonas	Bacteroidetes	3.13%	Sphingomonas	Proteobacteria	2.48%	Pseudomonas	Proteobacteria	3.85%
8	Streptococcus	Firmicutes	2.91%	Desulfobulbus	Proteobacteria	2.43%	Burkholderia	Proteobacteria	2.37%	Tolumonas	Proteobacteria	1.29%
9	Intestinimonas	Firmicutes	2.80%	Desulfomicrobium	Proteobacteria	2.19%	Mesorhizobium	Proteobacteria	2.32%	Klebsiella	Proteobacteria	1.03%
10	Psychrobacter	Proteobacteria	2.76%	Thiocystis	Proteobacteria	2.08%	Conexibacter	Actinobacteria	1.85%	Thauera	Proteobacteria	0.85%
11	Roseburia	Firmicutes	2.50%	Allochromatium	Proteobacteria	1.96%	Methylobacterium	Proteobacteria	1.71%	Parabacteroides	Bacteroidetes	0.76%
12	Oscillibacter	Firmicutes	2.48%	Ruminiclostridium	Firmicutes	1.69%	Corynebacterium	Actinobacteria	1.56%	Streptococcus	Firmicutes	0.63%
13	Lachnoclostridium	Firmicutes	2.10%	Methanocorpusculum	Euryarchaeota	1.55%	Amycolatopsis	Actinobacteria	1.47%	Flavobacterium	Bacteroidetes	0.59%
14	Ruminococcus	Firmicutes	1.66%	Geobacter	Proteobacteria	1.54%	Pseudonocardia	Actinobacteria	1.41%	Enterobacter	Proteobacteria	0.56%
15	Eubacterium	Firmicutes	1.65%	Marichromatium	Proteobacteria	1.36%	Lysobacter	Proteobacteria	1.29%	Sebaldella	Fusobacteria	0.53%
16	Corynebacterium	Actinobacteria	1.60%	Azoarcus	Proteobacteria	1.34%	Sphingopyxis	Proteobacteria	1.26%	Candidatus Cloacimonas	Candidatus Cloacimonas	0.51%
17	Odoribacter	Bacteroidetes	1.51%	Desulfovibrio	Proteobacteria	1.02%	Variovorax	Proteobacteria	1.23%	Citrobacter	Proteobacteria	0.50%
18	Escherichia	Proteobacteria	1.44%	Acidovorax	Proteobacteria	0.87%	Xanthomonas	Proteobacteria	1.22%	Alicycliphilus	Proteobacteria	0.48%
19	Alistipes	Bacteroidetes	1.17%	Sulfurimonas	Proteobacteria	0.81%	Rhizobium	Proteobacteria	1.13%	Alistipes	Bacteroidetes	0.48%
20	Lactobacillus	Firmicutes	1.08%	Rhodobacter	Proteobacteria	0.78%	Pseudoxanthomonas	Proteobacteria	1.08%	Peptoclostridium	Firmicutes	0.47%

Top twenty most abundant genera constituted 76, 62, 50 and 62% of microbiota of fecal composite, catch basin, soil and urban sewage influent samples respectively

The catch basin water community was dominated by Proteobacteria (67.4%), Actinobacteria (9.3%), Firmicutes (7.9%), Bacteroidetes (5.9%), Euryarchaeota (3.3%) and Spirochaetes (3.3%), accounting for 97% of prokaryotic microbiota reads (Fig. 1). Bacterial classes ɣ-proteobacteria and β-proteobacteria were abundant (Fig. 2) and constituted 45% of the prokaryotic reads, while Rhodocyclaceae and Moraxellaceae were the most abundant families in CB. Within these families, *Thauera* and Psychrobacter were the most abundant Proteobacterial genera in catch basin samples (Table 1). Psychrobacter are salt-tolerant, chemoheterotrophic, cold-adapted bacteria, which oxidize ammonia in high concentration under saline conditions [26]. Species from genus *Thauera* are frequently found in wet soil and polluted freshwater and have been considered important for industrial wastewater treatment systems as they play a key role in refractory aromatic hydrocarbon (e.g., indole and toluene) degradation under anaerobic and denitrifying conditions [26, 27]. *Thauera* were also observed in sewage influent. Occurrence of species from this genus in these polluted waters indicates the potential presence of aromatic hydrocarbons in these environments and as a result these functional species are of great significance for wastewater management.

The soil microbial community was predominated by Proteobacteria (60.3%) and Actinobacteria (35.2%), constituting 95.5% of the prokaryotic microbiota (Fig. 1). North American and European agroecosystems studies have also identified a high abundance of Proteobacteria and Actinobacteria associated with rhizosphere and rhizoplane [28, 29]. Wang et al. [30] have reported a 27 and 14% abundance of these two phyla respectively, in Chinese soils, followed by Acidobacteria (14%), Chloroflexi (8%) and Firmicutes (6%). In our soil samples, Bacteroidetes was the third most abundant phylum (1.6%), whereas Acidobacteria, Chloroflexi and Firmicutes were only present at 0.45, 0.41 and 0.13%, respectively. Lower abundance of Acidobacteria, and higher abundance of Proteobacteria, Actinobacteria, Firmicutes and Bacteroidetes has been associated with healthy agricultural soils with higher available phosphorus content [30]. Soil microbial communities can be highly diverse due to heterogeneity of soils, manure application as well as the nature of the rhizosphere [31]. In our soil samples, plant-associated species belonging to family Rhizobeaceae (α-Proteobacteria) were most prevalent (Table 1). Healthy soils generally have higher abundances of beneficial microbes including nitrogen-fixing and plant growth-promoting bacteria [32]. Interestingly, in present study, the soil collected 6 months after manure application had a higher number of Bacteroidetes (> 5 fold) and Euryarchaeota (> 3 fold) compared to non-manured and not recently manured fields. This likely reflects the presence of residual fecal bacteria from manure. Lupwayi et al. [33] also reported a higher proportion of Bacteroidetes in soils receiving composted beef feedlot manure in southern Alberta. While acknowledging the low number of soil samples originating from two agricultural fields in the vicinity of feedlot C over two years, inclusion of these samples in the analysis presents a snapshot of the influence of the feedlot manure on the soil microbiota and resistome.

Proteobacteria (83.5%), Bacteroidetes (10.4%) and Firmicutes (3.8%) represented the majority of sewage microbes with *Acinetobacter* (29%) and *Aeromonas* (16%) being the most abundant of the Proteobacteria. Others have found Proteobacteria to be among the most abundant bacteria in urban wastewater followed by Bacteroidetes and Firmicutes [34]. *Acinetobacter johnsii* and *Acinetobacter baumannii* accounted for the majority of the *Acinetobacter* identified. The former species rarely causes human infections, whereas the latter is an emerging hospital pathogen. In addition to being frequently recovered from patients during hospital outbreaks, *A. baumannii* have been reported in untreated as well as in biologically or chemically treated hospital and municipal wastewaters [35–38]. Our normalized species richness data indicated that SI harbored on average 2000 or more *A. baumannii* sequence reads as compared to FC, CB and soil (only 4, 15 and 1 respectively; Additional file 1) This suggests that the risk to human health from *A. baumannii* is far greater with SI than with the other environmental samples examined. In addition to *Acinetobacter* spp., the most abundant bacterial taxa detected in SI by others are Campylobacteraceae (*Arcobacter* spp.), Aeromonadaceae and Carnobacteriaceae [39–42]. Consistent with these studies *Arcobacter* and *Aeromonas* were among the most abundant genera in SI samples in our study, followed by *Acinetobacter*. Among *Aeromonas* spp. *A. hydrophila*, *A. media*, *A. veronii*, *A. salmonicida*, and *A. schubertii* were prevalent in SI. Most of these species are emerging human pathogens and have been associated with gastroenteritis, wound and soft tissue infections, necrotizing fasciitis, urinary tract infections, pulmonary infections in cystic fibrosis, and septicemia [43, 44]. *Aeromonas* spp. produce an array of virulence factors including cytolytic toxins with hemolytic activity and enterotoxins. Prevalence of these pathogens in FC, CB and soil was negligibly low as compared to SI.

**Fig. 3:**
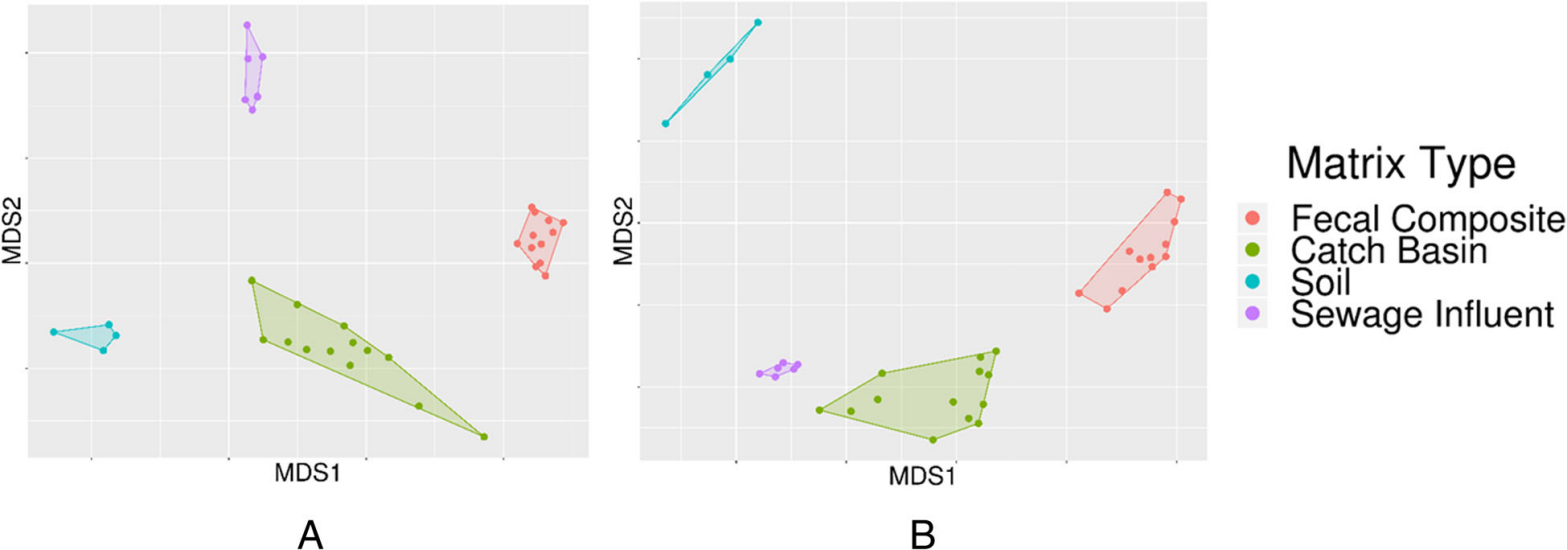
Comparative microbiota and resistome compositions of various sample types. Non-metric multidimensional scaling (NMDS) ordination plot indicate microbiota and resistome composition differences between fecal, catch basin, soil and wastewater (SI) samples at (**a**) genus (ANOSIM *P* = 0.001, ANOSIM R = 0.9804) and (**b**) AMR group (ANOSIM P = 0.001, ANOSIM R = 0. 9767) levels

Although 793 of the total 816 prokaryotic genera detected across all samples were represented in all sample types, their relative distribution was very unique between matrices (Fig. 2; Additional file 1). The non-metric multidimensional scaling (NMDS) plot formed distinct sample type-specific clusters (Fig. 3) with significant separation at all taxa levels (ANOSIM R: 0.9–0.98, *P* < 0.05; Fig. 3). As expected, the distinct microbial composition of each sample matrix appears to be a reflection of the unique composition of nutrients, physical, physicochemical and other biotic and abiotic factors within each niche.

**Fig. 4:**
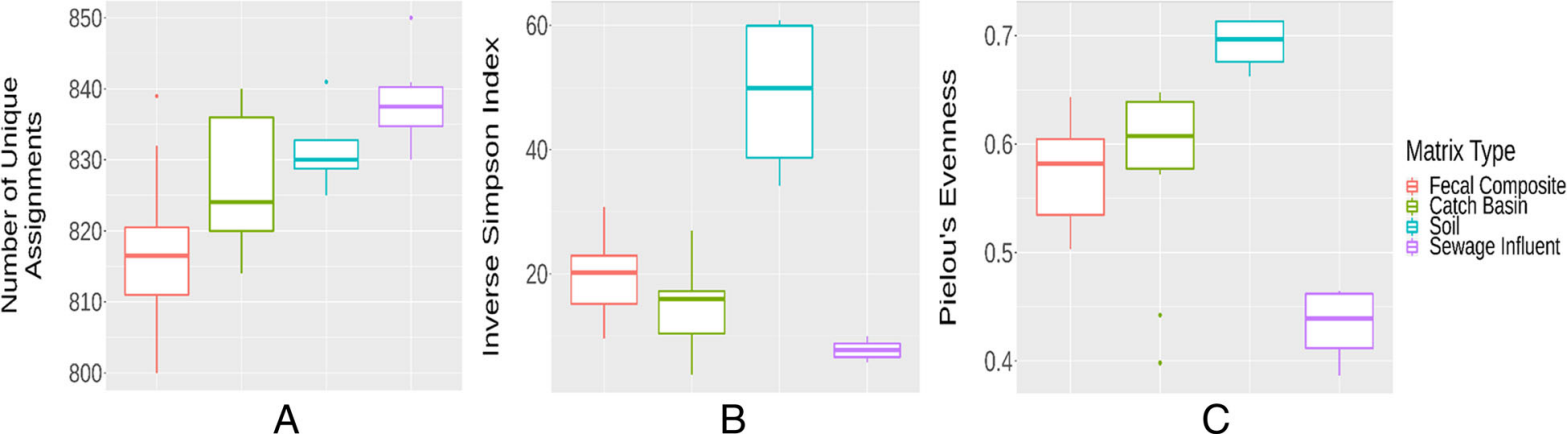
Quantitative comparisons of microbiota between various sample types. Richness (**a**) as indicated by number of unique taxa (genus discovery) assignments, α-diversity (**b**) as measured through inverse Simpson index, and evenness (**c**) of microbiota as Pielou’s evenness index at the genus level among various sample matrices are depicted by box-and-whisker plots. Boxes represent the interquartile ranges (upper line is the 75% quantile, and the lower line is the 25% quantile), the lines inside the boxes are the medians, the whiskers span the range of the 25% quantile or the 75% quantile plus 1.5 times the interquartile range, and dots are outliers

The SI microbiome exhibited the highest richness of microbial genera as indicated by the number of unique taxonomic (genus) assignments corresponding to discovery of new species, but the lowest α-diversity and evenness as depicted by low inverse Simpson and Pielou’s evenness indexes respectively, across all sample types (Fig. 4). Wastewater biosolids are a rich source of nitrogen, phosphorus, potassium and organic matter as well as micro-nutrients [45]. This nutrient-rich environment may allow certain resident bacteria to thrive and therefore promotes richness over diversity. Although the median α-diversity of phyla was higher for fecal samples than for any other matrices, soil had the largest (*p* < 0.05) median α-diversity at the lower taxonomic ranks.

### Distinct resistome composition of each sample matrix with predominance of tetracycline resistance in the beef production system

**Fig. 5:**
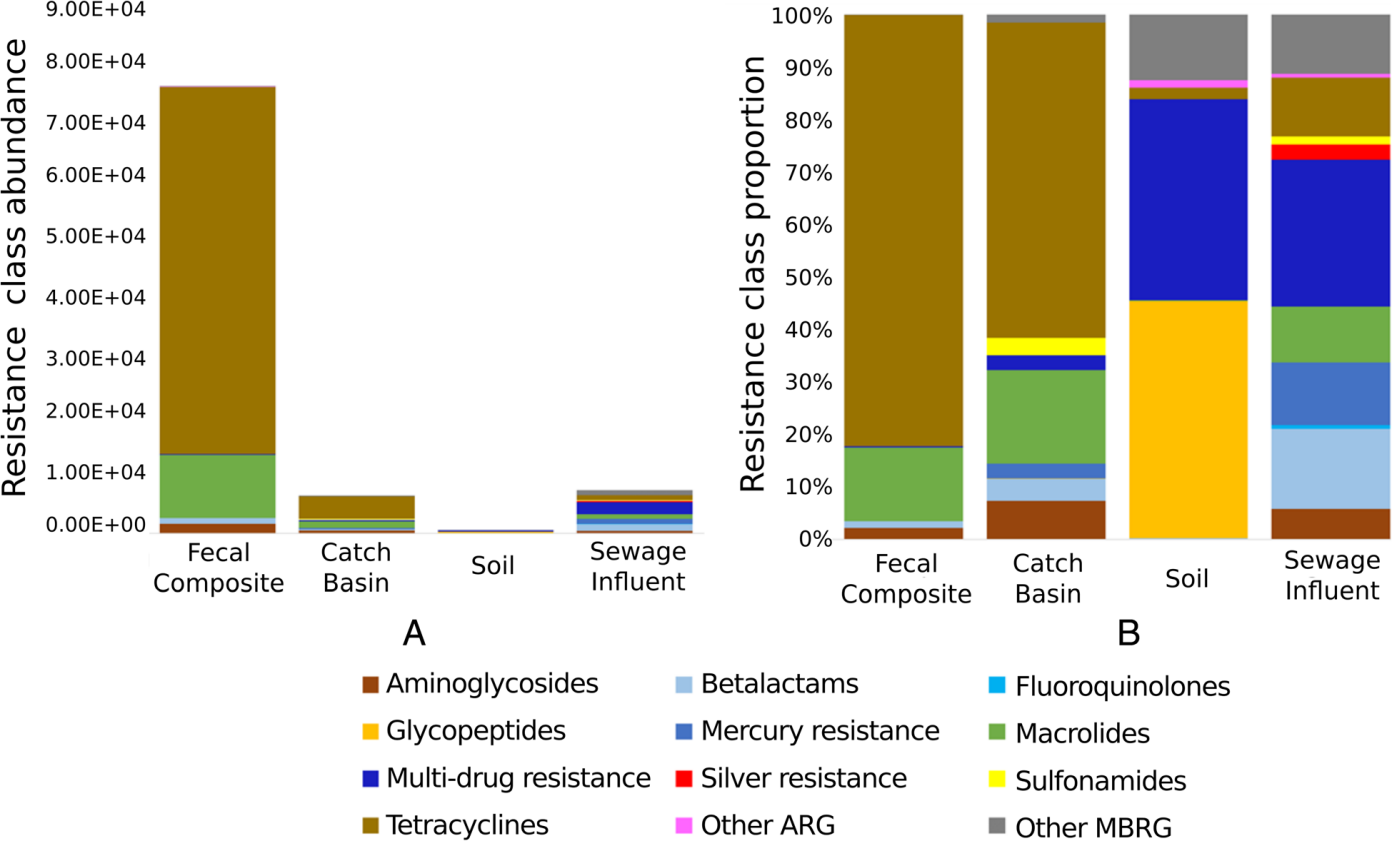
Abundance and relative proportion of antimicrobial resistance gene (ARG) and metal and biocide resistant gene (MBRG) classes in different sample types. Abundance (**a**) is a measure of read counts aligning to ARG-MBRG database and normalized across samples whereas proportion (**b**) indicates percentage of each class in a sample type. The category ‘Other’ includes the rest of the low abundance ARG/MBRG classes for each sample type

Across all samples, ~ 0.12% of total reads aligned to 35 mechanisms of antimicrobial resistance (AMR), coding resistance to 15 classes of antimicrobials, and ~ 0.04% of all reads corresponded to 15 classes of metal and biocide resistance (MBR) spanning 32 mechanisms. The proportion of AMR-MBR associated raw reads to the corresponding total reads was highest in conventional FC (0.25%) followed by SI (0.12%), CB (0.03%) and soil (0.002%), indicating a high prevalence of resistance genes in bovine feces. The proportion of AMR-MBR associated reads to the corresponding prokaryote-microbial reads was highest in conventional FC (11.3%) followed by CB (0.8%), SI (0.5%) and soil (0.07%) indicating that a higher fraction of bacteria and archaea in bovine feces harboured ARGs compared to other sample types. Comparison of normalized data across all samples also supported the larger abundance of ARG-associated reads in FC compared to soil, CB and SI (Fig. 5).

At the class level, tetracycline resistance was the most prevalent (82%) in FC followed by macrolide (14%), aminoglycoside (2.2%) and β-lactams (1.3%), respectively. Sequence reads aligned to 120 ARG and MBR gene (MBRG) groups collectively, belonging to 41 mechanisms within 18 classes. The tetracycline resistance ribosomal protection protein mechanism was most abundant (81%) predominantly represented by: TETQ,> TETW,> TET40,> TETO,> TET32 > TET44. Other tetracycline resistance genes including TET(X, M, A, B, G, 36, Z) were also present, but in lower abundance. Resistance to macrolides followed tetracycline resistance abundance, conferring lincosamide nucleotidyltransferases and efflux pump genes belonging to the LNUC and MEFA groups, respectively (Additional file 1). Previous studies reported a high prevalence of tetracycline resistance genes in cattle feces, with ~ 98% of reads aligning to ribosomal protection proteins represented in TETQ and TETW groups [46–48].

Overall, the CB resistome was represented by 84 ARG and MBRG groups. Similar to FC, in the CB resistome tetracycline resistance (59%) was the most abundant followed by resistance to macrolide (17.5%), aminoglycosides (7.2%) β-lactams (4.2%), sulfonamides (3.3%), mercury (2.8%) and multidrug resistance (MDR; 2.8%) (Fig. 5). This likely reflects the surface runoff of manure-associated tetracycline resistant ARB from feedlot pen floors into the catch basins. Miller et al. [49] quantified a runoff depth of 54 mm during a major rainfall event at a southern Alberta feedlot. Feedlots A, B, C and D shared 24, 31, 28 and 38 ARG groups between FC and their associated CB, respectively. The shared ARG groups were members of the tetracycline, macrolide and aminoglycoside resistance classes (Additional file 2). Among the tetracycline resistance groups, TETQ, TETM, TETW, TET36, TETT and TET44 were most prevalent. However, the relative abundance profile of these ARG classes differed between CB and FC reflecting the niche specificity of bacteria harboring these ARGs, considering that Proteobacteria were predominant in the CB microbial community as compared to Firmicutes and Bacteroidetes in FC. Among macrolide resistance ARG groups, MEFA, MEFB and MSR were more abundant in CB. Interestingly, MEFB was not detected in FC, but was present in SI samples. This gene has been found to be generally hosted by Proteobacteria [50], whereas MEFA and MSR genes have been associated with a wide variety of enteric bacterial phyla including Proteobacteria, Bacteroidetes, Actinobacteria and Firmicutes [51]. The high relative abundance of these genes could reflect their common presence in enteric bacteria, and/or due to co-selection with other ARGs as many tetracycline ARGs are linked to macrolide ARGs through common mobile genetic elements [52].

In North America, the use of in-feed tetracycline and macrolides to prevent liver abscesses and other bacterial diseases is a common management strategy in beef cattle production. Macrolides are also used to treat and manage Bovine Respiratory Disease (BRD). Conventional feedlots in the present study administered ionophores in combination with chlortetracycline or tylosin in-feed on a daily basis throughout the feeding period. Occasionally, therapeutic doses of antimicrobials were also administered to clinically ill cattle within a pen. It is acknowledged that the physical presence of a resistance gene may not always be interpretable as functional presence in the absence of gene expression data. However, the presence of an abundant gene is generally associated with some degree of its functional expression within a particular environment. The high prevalence of both tetracycline and macrolide resistance gene classes in FC and CB, therefore is likely a reflection of the ubiquitous use of these antibiotics in beef production [53, 54].

Soil samples originating from agricultural fields adjacent to feedlot C had a small and unique resistome with only 9 ARG groups belonging to 6 classes and did not align with the feedlot resistome (Fig. 5; Additional file 1). Tetracycline ARG TETL was only found in recently manured soil. Compared to soil, this ARG group had a 9–17 times lower prevalence in FC and CB and was completely absent in SI. It may be that TETL harboring bacterial species from manure survived better in soil compared to other tetracycline ARG carrying bacteria. Tetracycline was the most widely used antibiotic class in the feedlots enrolled in this study. Glycopeptide resistance associated genes were present across all soil samples, but were absent from any other sample type. Specifically, VanO-type regulators (VANRO) [55] were the only glycopeptide-related genes detected in soil samples. The *vanO* operon initially identified in *Rhodococcus equi* [55], harbors a *vanHOX* resistance gene cluster transcribed convergent to that of the *vanS-vanR* two-component regulatory system. The *vanO* locus in *Rhodococcus equi* exhibits similarity to genera *Amycolatopsis* and the nitrogen fixing, root nodule-forming *Frankia* [55] and to the teicoplanin producer *Actinoplanes teichomyceticus* [56]. The *Amycolatopsis* and *Actinoplanes* were among the most prevalent genera in soil samples from our study (Table 1). Other than *vanO*-type regulators no other vancomycin resistance operon-associated reads (Vancomycin D-alanyl-D-alanine dipeptidase and/or ligase etc.) were detected, which may be due to low homology or absence of the *vanO* operon associated genes in soil bacteria. The second most abundant ARGs in soil were multidrug resistance (MDR) efflux pump coding genes. The organisms with the largest number of MDR pumps are in fact found in the soil or in association with plants [57]. Along with their potential roles as multidrug efflux pumps, these are important for detoxification of intracellular metabolites, bacterial virulence in both animal and plant hosts, cell homeostasis, and intercellular signal trafficking [58]. Therefore, bacteria harboring MDR pumps are not always associated only with high antibiotic load environments.

The SI from two urban municipalities in Southern Alberta exhibited similar resistome composition. Across all sample matrices SI had the largest number of ARG groups (229) belonging to 28 classes of ARGs and MBRGs. The most prevalent resistance classes in SI included multi-drug resistance (28%), β-lactam (15.28%), mercury (11.83%), tetracycline (11.16%) macrolide (10.72%) and aminoglycoside resistance (5.78%) (Fig. 5). Historically, mercury contamination of wastewater occurs from a variety of sources including dental practice wastes, lawn fertilisers, landfill leachate, paints, domestic waste inputs, groundwater infiltration and storm water drainage. Of the 2000 tonnes per year of global atmospheric mercury that is discharged into the air and water from anthropogenic sources, Canada’s atmospheric mercury share account for <0.5% of the world’s emissions (https://www.canada.ca/en/environment-climate-change/services/pollutants/mercury-environment.html).

Among β-lactam ARGs, cephalosporin resistance groups OXA and CTX were predominant, with 8 fold more richness of OXA in SI compared to CB, and its complete absence in FC and soil. Conversely, CTX was 71 fold more abundant in SI compared to FC and absent in CB and soil (Additional file 1). QNRD, a plasmid-mediated quinolone resistance (PMQR) gene group was only present in SI, likely reflecting its use in human medicine. Among all sample types, only the SI resistome contained a large variety of metal and biocide resistance genes (Additional file 1). Recently, Gupta et al. [42] reported a similar relative abundance of ARGs and a high prevalence of heavy metal resistance genes (HMRGs) in samples from a wastewater treatment plant.

Thirty four ARG groups belonging to tetracycline (TET32, TET40, TET36, TETA, TETG, TETM, TETO, TETS, TETQ, TETW, TETX,), macrolide (ERMA, ERMB, ERMF, ERMG, LNUC, MEFA, MEL, MPHB, MPHE, MSR, MSRD), aminoglycoside (ANT6, ANT9, APH3’ APH3”, APH6, SAT, ANT3”), class A beta lactamase (CFX & CARB), sulfonamide (SULII), mercury resistance (MERA), and drug and biocide small multidrug resistance (SMR) efflux pump (qacEΔ1) were shared in FC & CB and SI sample groups at varying abundances (Additional file 1). For tetracycline resistance TETQ, TETW, TET40, TETO, TET32 and TET44 were among the most abundant tetracycline ARG groups in beef production. TETQ, TETW, TET40, TETO and TET32 have also been identified among the most prevalent groups in fecal samples collected from humans in China, Denmark and Spain [59, 60], suggesting their high abundance in both cattle and human microbiota. Studies across diverse agricultural ecosystems have also demonstrated the ubiquity of tetracycline resistance genes [61, 62].

Sewage wastewater is an effective source of fecal bacteria and provides a unique opportunity to monitor fecal microbes from large human populations without compromising privacy [63]. Wastewater treatment plants are considered hotspots of ARB and ARGs [15, 64, 65], as they receive wastewater from households and hospitals where antimicrobials are administered. The persistent selective pressure posed by sub-inhibitory concentrations of antimicrobial residues in wastewater combined with the high density [17] and diversity [66] of microorganisms could promote horizontal transfer of ARGs and HMRGs [67–69]. Co-selection of ARGs and HMRGs in SI [70, 71] is favoured when these genes are carried on the same mobile genetic element [72]. Furthermore, leachate from wastewater sludge disposed of in landfills may promote the spread of ARGs into sub-soils and ground water [73].

**Fig. 6:**
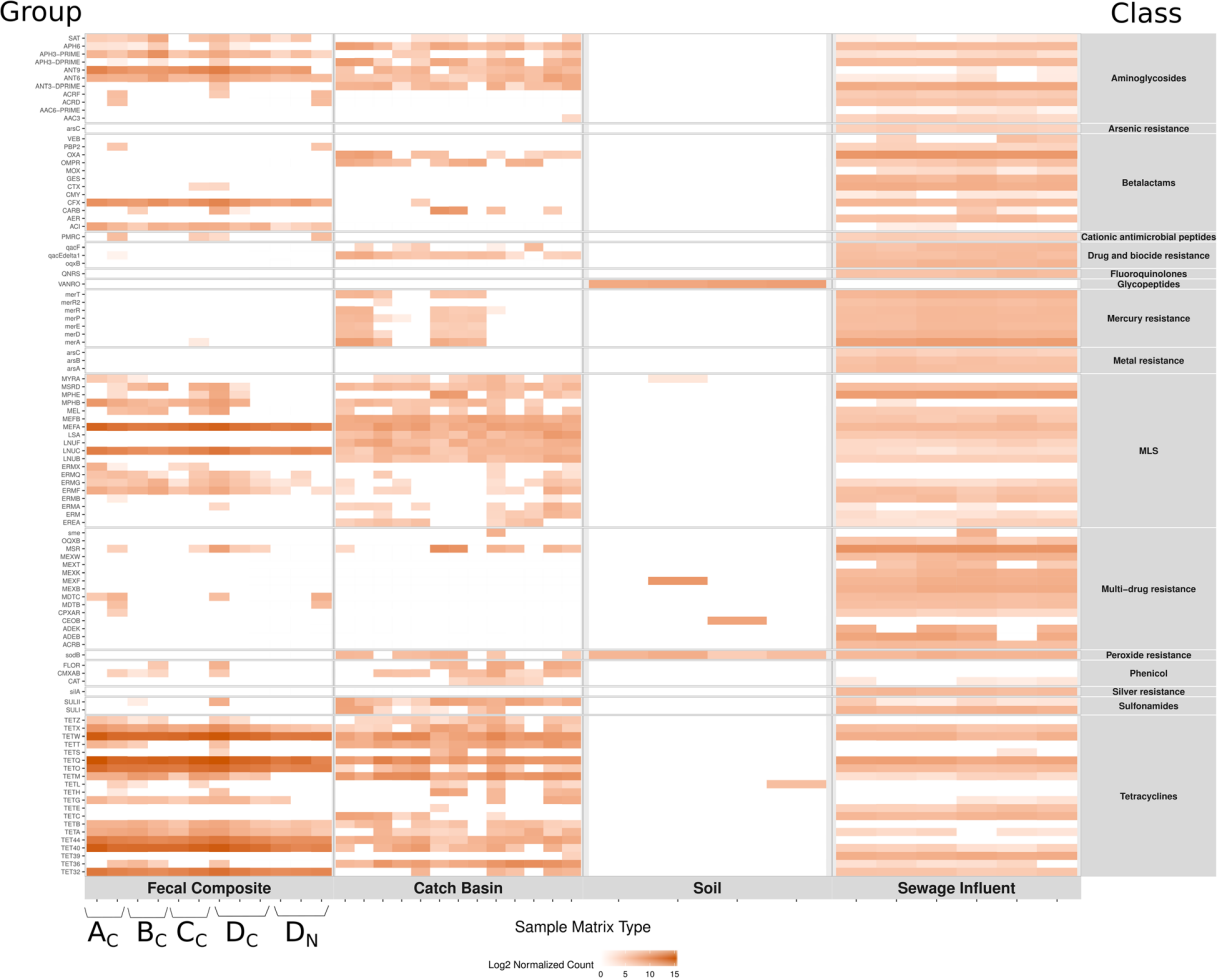
Heat map of prevalent antimicrobial resistant gene groups across all samples grouped by antimicrobial resistance class. As described in methods section, fecal composite samples were obtained from 4 feedlots **a**, **b**, **c** and **d**. The subscript letters _C_ and _N_ denote conventional and natural practices, respectively

A heat map of prevalent ARGs groups across all samples grouped by AMR classes (Fig. 6) indicated that the majority of AMR/MBR classes represented in FC, CB and SI resistome were absent in soil. Tetracycline, β-lactam and multidrug efflux ARGs were present among all sample types, whereas ARGs for fluoroquinolones, fosfomycin and metronidazole were only present in SI (Additional file 1), suggesting that use of these antimicrobials in humans selected for these genes. The NMDS analysis showed that the resistome from different sample types differed at the AMR gene group (ANOSIM *P* = 0.001, ANOSIM R = 0. 98) level (Fig. 3B) and all other levels of ARG categories (ANOSIM *P* < 0.05, R: 0.92–0.98) confirming the uniqueness of resistome in each sample type. Across sample types, 5, 9, 98 and 5 resistance gene groups were uniquely present in FC, CB, SI and soil respectively (Fig. 6; Additional file 2). In addition to the microbial source and the microbial niche specificity in different environments the distinct resistome composition of each sample matrix could also be a reflection of the specific antimicrobial residues in each environment. Recent studies have identified a link between community structure and antibiotic resistance gene dynamics [74]. Future metagenomics-based microbiome and resistome studies that include bacterial genome assemblies from deep metagenomics sequencing data will shed light on the association of ARGs with their host bacteria.

**Fig. 7:**
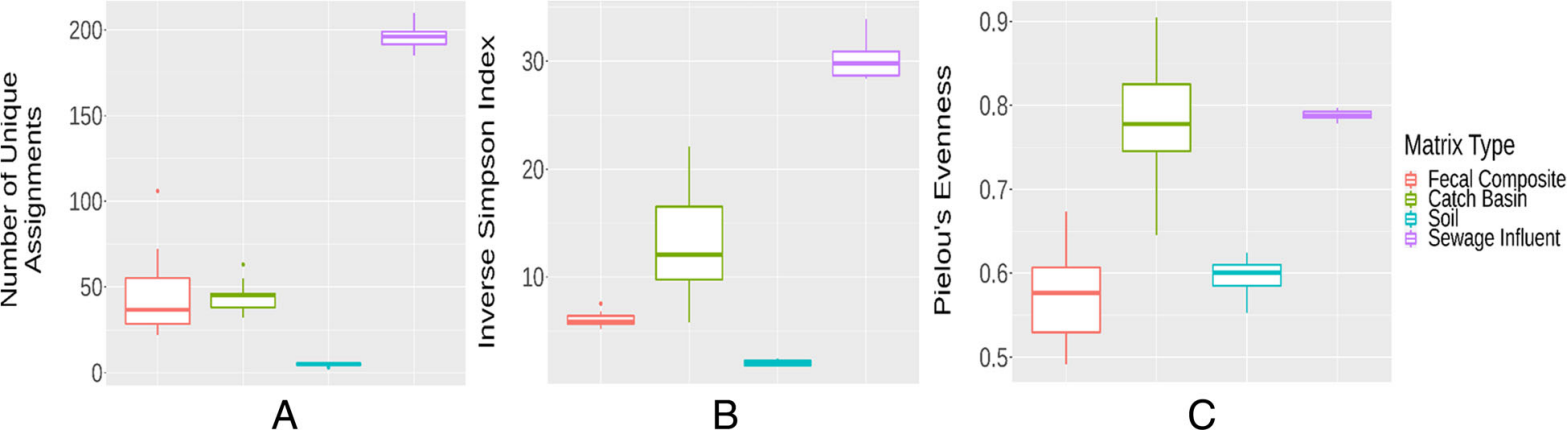
Quantitative comparisons of resistome between various sample types. Richness (**a**) as indicated by number of unique gene groups (gene group discovery) assignments, α-diversity (**b**) as measured through inverse Simpson index, and evenness (**c**) of resistome as Pielou’s evenness index at the resistance gene group level among various sample matrices are depicted by box-and-whisker plots. Boxes represent the interquartile ranges (upper line is the 75% quantile, and the lower line is the 25% quantile), the lines inside the boxes are the medians, the whiskers span the range of the 25% quantile or the 75% quantile plus 1.5 times the interquartile range, and dots are outliers

The SI wastewater resistome exhibited the highest richness of ARG mechanism types among sample types (Fig. 7). In addition to having high richness, SI contained the most diverse and even resistome among all sample types as indicated by high inverse Simpson index of α-diversity and Pielou’s evenness index (Fig. 7B), which reflects the diverse classes of antimicrobials used in human medicine [75] as compared to those used in cattle. After ionophores, tetracycline and macrolides are among the most frequently used antimicrobials in livestock [76, 77].

### Natural feedlot FC samples harboured relatively similar microbiota but smaller resistome compared to conventional samples

The microbial composition of fecal samples from ‘natural’ and ‘conventional’ beef production systems had comparable richness, diversity, and similar prevalence of microbial phyla. The exception was that the composition of natural FC microbiota had a lower abundance of two bacterial (Bacteroidetes, Spirochaetes; log FC values − 0.7 and − 2.3 respectively; *p* < 0.05) and one archaeal (Euryarchaeota; log FC value − 3.8; *p* < 0.001) phyla in natural, compared with conventional FC. A 17-fold increase in the methanogenic archaeal genus *Methanobrevibacter* (Phylum Euryarchaeota) was observed in the samples originating from conventional pens as compared to the natural pens (Additional file 1). Considering that the animal diets between the natural and conventional feedlot practices were similar, these differences in fecal microbiota may be related to antimicrobial use. Given the small number of samples compared between natural and conventional feedlots, further studies are needed to more thoroughly investigate this phenomenon.

**Fig. 8:**
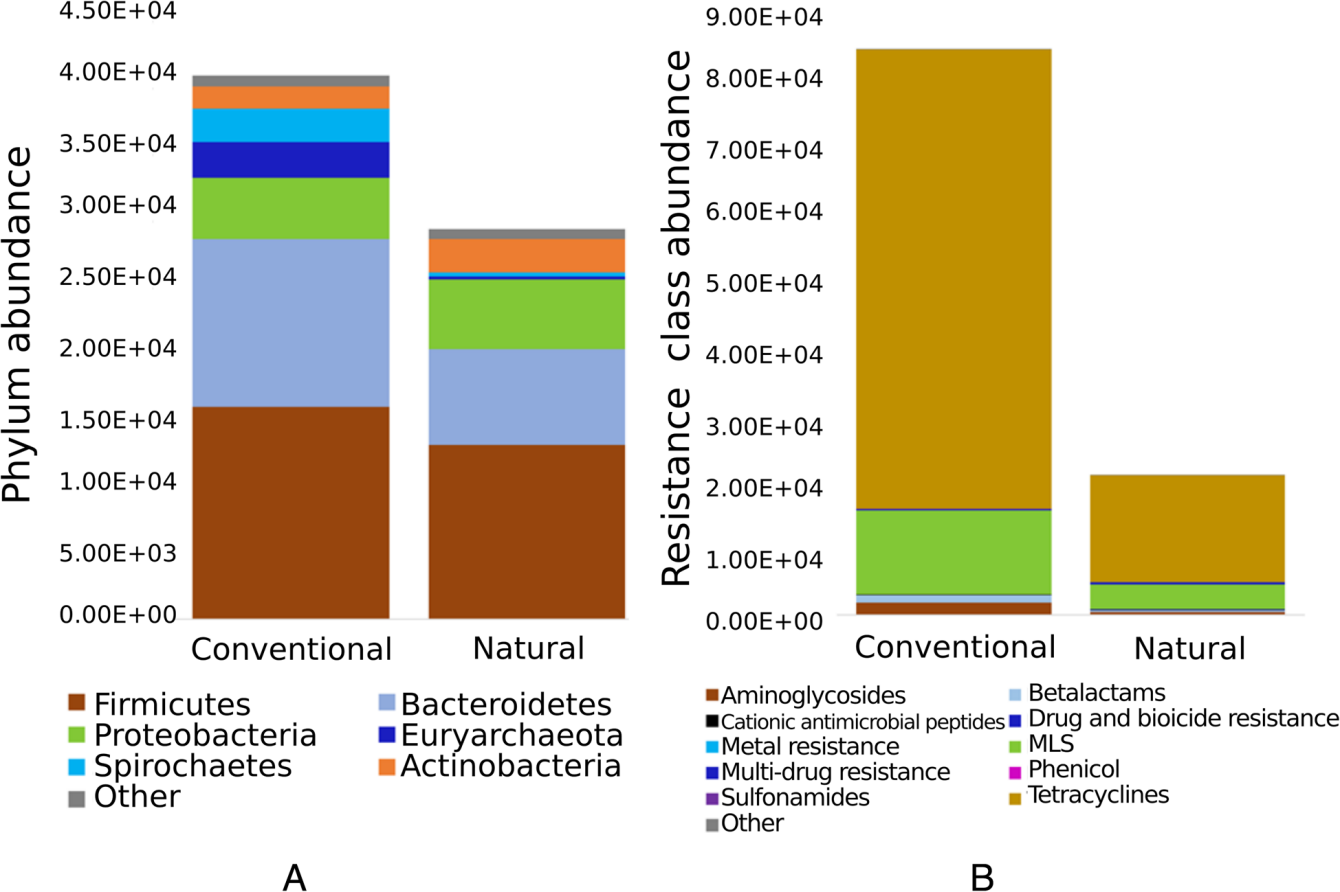
Comparisons of microbiota and resistome between samples from conventional and natural feedlot pens. Relative abundance of microbial phyla (**a**) and antimicrobial resistance classes (**b**) among fecal composite samples from conventional and natural feedlot pens are indicated as read counts on the X-axis

The proportion of AMR-MBR associated raw reads to the corresponding total reads for feedlot D conventional FC samples was higher (0.23%) compared to natural FC samples (0.09%) indicating high prevalence of resistance genes in bovine feces. The average number of ARG-associated reads identified was higher for the conventional FC compared to natural FC (Fig. 8). This trend was observed across the top three abundant ARG classes including tetracycline, macrolide and aminoglycoside (p < 0.05). Regardless of higher ARG abundance in conventional samples, diversity of resistomes between natural and conventional pen samples was similar (Additional file 1). Prior studies have concluded no correlation between the presence of antimicrobial resistance genes in the gut microbiota and the administration of antibiotic feed additives [78–81]. However, in contrast to our study, most of these studies either did not quantify comparative prevalence of ARGs in production systems managed with and without using antimicrobials or their comparative investigation was limited to a few bacterial species and ARGs. Single-colony subcultures do not recover the actual AMR reservoir of a microbial community.

Phenicol and sulfonamide were the only resistance classes absent in the natural samples. Other groups belonging to tetracycline (TETA, TETB, TET32, TETW, TET40, TET44, TETO, TETQ, TETX), macrolide (MEFA, LNUC), aminoglycoside (APH3’, ANT6) and β-lactams (CFX, ACI) resistance were present in both natural and conventional FC, whereas tetracycline (TETH, TET36, TETZ, TETS, TETT), macrolide (APH6, MPHE, MPHB, MSRD ERMA, MPHE, MEL, ERMR, ERMC, ERMT), aminoglycoside (ANT3”), β-lactamase (CARB), phenicol (FLOR, CMXAB) and sulfonamide (SULII) were absent in natural samples, but were present in at least one of three conventional samples. The ARG groups MSR and TETM belong to macrolide and tetracycline drug classes respectively, and were present in all conventional FC pen samples from feedlot D, but were absent in all natural pen samples. Assuming that the presence of a gene means that it is being expressed, their presence may be associated with the use of these drug classes in the conventional feedlot. Genes belonging to this family have been shown to be associated with transposons and integrative conjugative elements [82, 83], which may contribute to their ubiquitous prevalence through intra- and inter-species mobility under the added selective pressure of antimicrobial use. Considering that ARGs are ancient [84] their diverse presence in natural production systems is not surprising. The occurrence of certain ARGs within bacterial populations is likely a reflection of their association with fitness traits that enable bacteria to persist within a particular environment. While antibiotic resistance and its spread by horizontal gene transfer are ancient mechanisms, the rate at which these processes occur and the proliferation of certain ARG-harboring bacteria has increased tremendously over the last decades due to the selective pressure exerted through anthropogenic administration of antimicrobials. We argue that a holistic approach of identifying ARGs and microbiota, and quantitating their prevalence as undertaken in our study is necessary for informing surveillance and to understand the evolution and transmission of AMR in an environmental spectrum.

## Conclusions

Consistent with its abundant use in feedlots, tetracycline resistance was predominant in the beef production system followed by macrolide resistance. Regardless of possessing a comparable composition of microbiota, fecal samples collected from cattle raised without antibiotics exhibited a smaller resistome as compared to fecal samples collected from conventionally raised cattle. This study enhances our understanding of the microbial composition and the occurrence of ARGs and identifies common elements between those components of the environmental spectrum and indicates a distinct separation of associated microbial communities. The specific resistance profiles across various sample matrices were dependent upon the microbial community composition as well as differences in the nature and prevalence of drug, metal and biocide contaminants.

## Methods

### Sample collection, DNA isolation, quantitation and quality assessment

Composite fecal samples analyzed in this study (*n* = 12) were collected from four different beef cattle feedlots (A, B, C, D) within the province of Alberta Canada (sampling locations in Additional file 6: Fig. S1). Feedlot sampling was conducted from April – June 2014. The feedlots had operating capacities of ∼15,000–30,000 head of cattle. Production conditions were typical of western Canadian commercial feedlots, with animals housed in open-air, clay-floor pens arranged side-by-side with central feed alleys. Feedlot D had two separate wings for hosting natural (raised without antibiotics) and conventional (with antibiotics) cattle pens. Samples in Feedlot D were collected from both natural (*n* = 3) and conventional (n = 3) pens. The rest of the fecal composite samples (*n* = 6 of a total of 12) originated from conventional feedlots A, B and C (Supplementary data_3), where antimicrobials were used in a routine manner similar to the conventional wing in Feedlot D. Within a feedlot, samples were collected on the same day from pens containing 150–300 animals. Sampling procedures were reviewed and approved by the Lethbridge Research Centre Animal Care and Use Committee (AC# 14–0029), and were conducted according to the Canadian Council of Animal Care Guidelines. Each composite fecal sample comprised ~ 20 g aliquots collected from 20 individual fresh fecal pats within each pen. Fecal samples were thoroughly mixed, placed in 532 mL Whirl-Pak bags, flash frozen in liquid nitrogen and stored at -80 °C. The antimicrobials used in the sampled conventional feedlots are listed in Additional file 4. The in-feed antimicrobials (ionophores, chlortetracycline or tylosin) were administered to all cattle in the conventional feedlot throughout the feeding period with the therapeutic parenteral drugs administered to clinically ill cattle as required.

Natural resources legislation in Alberta stipulates that feedlots must have catch basins (also known as retention or runoff holding ponds) for containment of surface runoff water from pens or manure storage areas generated by rainfall or snowmelt. At each feedlot, surface water was sampled from a catchment basin adjacent to the sampled feedlot pens. Water samples (2, 3, 4 and 4 samples were collected from catch basins at feedlots A, B, C, and D respectively, *n* = 13) (Additional file 3). One liter of water was collected at a depth of 0.5 m into a 1.3 L polyethylene bottle attached to a telescopic pole. Water was collected from four different locations within the catchment basin and the samples were combined to generate a single composite sample which was immediately transferred to the lab on ice. To complement the cattle production and associated environmental sampling, two wastewater treatment plants in Southern Alberta (Additional file 1: Fig. S1) provided sewage influent samples (n = 6) to represent the urban element of the environmental spectrum. One liter of sewage influent water was collected from post-grit tanks of the wastewater treatment facility.

Catchment basin or sewage influent water samples (n = 13, up to 100 mL each) were filtered through 0.45 μm pore size nylon filters (MilliporeSigma, Etobicoke, ON, Canada) using a water filtration manifold and membrane filtration units (Pall Corporation Ltd. Mississauga, Canada). The membrane filter was aseptically removed from the filter base using sterile forceps and stored at − 20 °C in a sterile 5 ml OMNI Bead Ruptor tube (Cole-Parmer, Montreal, Canada) for later DNA extraction. If the membrane filter became plugged, samples were centrifuged at 10,000 x g in 50 mL tube to obtain a pelleted biomass for DNA extraction.

Composite core soil samples (*n* = 4) were collected from agricultural fields adjacent to feedlot C and included the following sample types: field with no history of manure application, from the same field as above but ~ 6 months after manure application, and from a field with a continuous history of manure application, but not within 1–2 year prior to sampling. Soil samples were collected twice over two years (see Additional file 3 for details). Soil sampling was carried out using a soil coring kit (5 cm diameter) to a depth of 10 cm and samples at 10 points along a 100 m transect were collected and pooled for each field to constitute a composite sample.

Metagenomic DNA isolation from the bovine fecal samples was performed as previously described [16]. The DNA was extracted from soil and pelleted biomass from water samples in a manner similar to feces, with the nylon filters subject to bead-beating and incubation steps at 70 °C [16]. The DNA concentrations were measured using the Quant-iT™ PicoGreen (Thermo Fisher Scientific, Mississauga,ON, Canada) and the DNA purity was determined by measuring the ratios of absorbance at 260/280 and 260/230 using a NanoDrop spectrophotometer (Thermo Fisher Scientific). The DNA extracts with a 260/280 ratio between 1.8–2.0 and a 260/230 ratio between 2.0–2.2 were regarded as pure. The presence of PCR-inhibitors was also evaluated by amplifying the 16S rRNA gene using the universal 16S rRNA gene primers 27F and 1492R [85] with undiluted and diluted samples [16].

### Metagenomic DNA sequencing and data processing

All library preparations, next generation sequencing and quality control steps were performed by the McGill University and Genome Quebec Innovation Centre (Montréal, QC, Canada). TruSeq DNA libraries were prepared and samples were run on an Illumina HiSeq2000 platform, with 4 samples multiplexed per sequencing lane to generate 2 × 100 base paired-end (PE) sequences [16]. As a quality control for cluster generation and sequencing, each HiSeq2000 sequencing lane was spiked with the PhiX174 sensu *lato* virus genomic DNA library at ~ 1% concentration of the total DNA loaded per lane.

Trimmomatic version 0.36 [86] was used to remove adapter contamination and low quality reads using the following parameters: trimming leading and the trailing low quality or N bases (below quality 3) from sequence reads; performing quality score filtering using a sliding window at every four bases with a minimum Phred score of 15; discarding sequences with < 36 nucleotides; removing adapters supplied in the TruSeq3 adapter sequence file using a maximum of 2 mismatches in the initial seed, and clipping the adapter if a match score of 30 was reached. Singleton reads, whereby the other pair was discarded were also included in downstream analysis.

### Determination of the taxonomic and ARG composition of microbiota

Taxonomic classification of microbiota and determination of AGR assignments for resistome analysis of the sequence data were performed using previous methods and parameters [16]]via a Galaxy Web server instance (https://galaxyproject.org/) supported by the National Microbiology Laboratory, Public Health Agency of Canada (PHAC NML Galaxy). The Kraken taxonomic classification tools (version 0.10.5 beta) and the resistome analysis tools were integrated in a workflow to obtain output for both the resistome and microbiome analyses (workflow details in Additional file 6: Fig. S2).

In that workflow, the trimmed paired reads that passed the quality assessment criteria from the pre-processing step with Trimmomatic were aligned to the genome of the enterobacteria phage phiX174 (GenBank accession NC_001422.1) using the minimum exact match (MEM) algorithm of the Burrows-Wheeler aligner (BWA) [87]. The sorted alignments were then processed with *samtools* [88] to filter out reads that did not map to the PhiX 174 bacteriophage genome. This was done using a flag value of 4 to extract the unmapped reads in binary alignment map (BAM) format. The paired reads that did not map to PhiX 174 bacteriophage were then extracted from the alignment using the *bamToFastq* tool of *BEDTools* [89]. The PhiX-filtered reads were then classified with Kraken v 1.2.3 [90] using the custom Kraken database bvfpa [16]. Kraken results were filtered using a confidence threshold of 0.05 to select for taxonomic assignments with high precision and sensitivity and thus high accuracy at the genus rank [http://ccb.jhu.edu/software/kraken/MANUAL.html; 16]. Resistome analysis was conducted in parallel with the taxonomic classification as follows: Trimmed paired reads were mapped to the ARG sequences in the MEGAREs database v1.01 [91] combined with a custom metal and biocide resistance (MBR) database (MegaBio; P.S. Morley’s lab; Additional file 5) using BWA-MEM v 0.7.17.1 [87] alignments in BAM format followed by conversion to sequence alignment map (SAM) format and post-processing with the Coverage Sampler tool (https://github.com/cdeanj/coveragesampler) using a 75% gene fraction threshold and other parameters [15].

### Data analyses

The microbiome and resistome data reports from individual samples were aggregated into corresponding matrices using R for downstream analyses. Microbiome and resistome matrices were normalized using the data-driven approach of Cumulative Sum Scaling normalization (CSS) with the metagenomeSeqR package [92]. This method calculates a scaling threshold that is the quantile after which the distribution of raw counts among samples is invariant. The method calculates the sum, up to and including that quantile threshold for re-scaling. In this study, a CSS normalization quantile threshold of 0.5 (the median) was used. The cumulative sum scaling method has been previously reported for normalization of comparative metagenomic sequencing data from various environments [93]. CSS has greater sensitivity and specificity compared to other normalization methods and it corrects the bias in the assessment of differential abundance introduced by total-sum normalization therefore improving sample clustering [94]. Other methods such as rarefaction analysis can lead to higher false discovery rate while comparing differentially abundant genes [95]. The exploratory analyses performed in this study included: relative abundance analysis for microbiome and resistome for all sample matrix types, assessment of α-diversity and richness for all sample types, ordination using nonmetric multidimensional scaling (NMDS), and comparative visualization of data with heatmaps and barplots. Observed richness, the Shannon’s and Inverse Simpson’s α -diversity indices, and Pielou’s evenness were calculated using functions of the vegan package version 2.5.1 [96] and their distributions were plotted for each sample type as box-and-whisker plots using ggplot2 [97]. Heatmaps were constructed using the log_2_ transformed CSS-normalized counts which were plotted using white to orange gradient scale.

A zero-inflated Gaussian (ZIG) mixture model was applied to evaluate differentially abundant features in the resistomes and microbiomes between sample matrix types. This model has been reported to increase sensitivity and specificity when working with datasets with high sparsity (abundance of zero counts). Ordination plots were generated using NMDS and statistical inference was made using the analysis of similarity (ANOSIM) with the vegan R package version 2.5.1 [96]. ANOSIM *R*-values ranged from 0 (total similarity) to 1 (total dissimilarity). The Kruskal–Wallis test [98] was performed to compare the distributions of richness and the Inverse Simpson’s indices of α--diversity for both ARGs and microbial taxa among the various sample types. Nemenyi post-hoc comparisons [99] were conducted for incidences where differences were declared significant at *P* < 0.05 as per the Kruskal-Wallis analysis. The R code for the data analysis is available at https://github.com/ropolomx/one_health_continuum.

## Additional files

Additional file 1: Microbiota and AMR stats – Ten Excel sheets describing raw and normalized read count data for microbiome and resistome of studied samples. (XLSX 3121 kb)

Additional file 2: Shared and Unique ARG groups – Two Excel sheets with lists of shared and unique ARG groups among various sample types. (XLSX 17 kb)

Additional file 3: Sample metadata and sequencing stats – Two excel sheets describing details of sample collection metadata and Illumina HiSeq read counts of studied samples. (XLSX 12 kb)

Additional file 4: Antibiotics used in feedlots – Excel sheet describing antibiotics used in the feedlots enrolled in present study and their mode of administration. (XLSX 9 kb)

Additional file 5: Excel file describing accession and annotation details of genes included in metal and biocide database, MegaBio. (XLSX 28 kb)

Additional file 6: **Figure S1.** Sampling locations in the province of Alberta, Canada. **Figure S2.** Galaxy workflow for antimicrobial resistance (AMR) and taxonomic profiling of metagenomics sequencing read data. (DOCX 1067 kb)

## Data Availability

All Illumina sequence read data from current study have been deposited to the NCBI database as Short Read Archive (SRA) under BioProject IDs PRJNA420682, PRJNA529711, PRJNA507800 and PRJNA482680. These data are publically available at https://www.ncbi.nlm.nih.gov/bioproject/.

## Abbreviations

AMR: Antimicrobial resistance
AMU: Antimicrobial use
ANOSIM: Analysis of similarity
ARB: Antimicrobial resistant bacteria
ARG: Antimicrobial resistance gene
BAM: Binary alignment map
CB: Feedlot catchment basin
CSS: Cumulative sum scaling normalization
FC: Composite fecal sample
HMRGs: Heavy metal resistance genes
MBR: Metal and biocide resistance
MBRG: Metal and biocide resistance gene
MDR: Multidrug resistance
MEM: Minimum exact match
NGS: Next generation sequencing
NMDS: Nonmetric multidimensional scaling
SI: Urban sewage influent
STP: Sewage treatment plant
