# Supplementary material for: Comparative diversity of microbiomes and Resistomes in beef feedlots, downstream environments and urban sewage influent
Source: BMC Microbiol. 2019 Aug 27;19:197. doi: 10.1186/s12866-019-1548-x (PMC6712873; doi:10.1186/s12866-019-1548-x)
Supplement: Supplementary file 6 — Figure S1. Sampling locations in the province of Alberta, Canada. Figure S2. Galaxy workflow for antimicrobial resistance (AMR) and taxonomic profiling of metagenomics sequencing read data. (DOCX 1067 kb) [file 12866_2019_1548_MOESM6_ESM.docx]

***Supplementary Material***

**Comparative Diversity of Microbiomes and Resistomes in Beef Feedlots, Downstream Environments and Urban Sewage Influent.**

Rahat Zaheer^1^, Steven Lakin^2^, Rodrigo Ortega Polo^1^, Shaun R. Cook^3^, Francis J. Larney^1^, Paul S. Morley^2^, Calvin Booker^4^, Sherry Hannon^4^, Gary Van Domselaar^5^, Ron R. Read^6^, Tim A. McAllister^1^*

^1^Agriculture and Agri-Food Canada, Lethbridge Research and Development Centre, 5403 1 Ave, Lethbridge AB, T1J 4P4, Canada

^2^Department of Clinical Sciences, Colorado State University, Fort Collins, CO 80523, USA.

^3^Alberta Agriculture and Forestry, 100, 5401 – 1^st^ Avenue South, Lethbridge AB, T1J 4V6, Canada

^4^Feedlot Health Management Services, Okotoks, AB, Canada

^5^National Microbiology Laboratory, Public Health Agency of Canada, 1015 Arlington Street, Winnipeg, MB, R3E 3R2, Canada

^6^Cumming School of Medicine, University of Calgary, 3280 Hospital Drive NW, Calgary, Alberta

* [tim.mcallister@canada.ca](mailto:tim.mcallister@canada.ca)

**
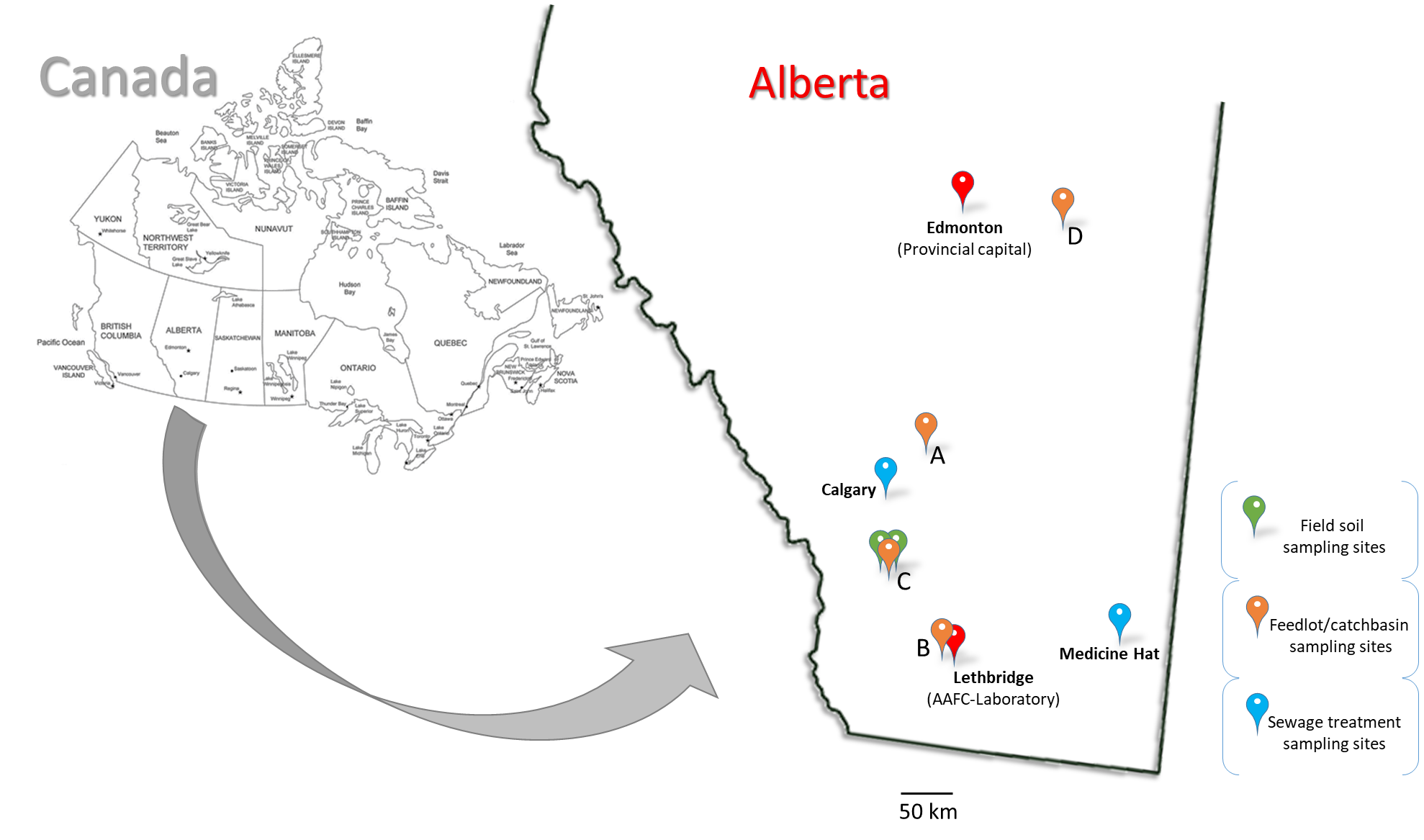
**

**Figure S1: Sampling locations in the province of Alberta, Canada.** Provincial capital (Edmonton) and the laboratory location (Lethbridge) are shown as reference points.

**
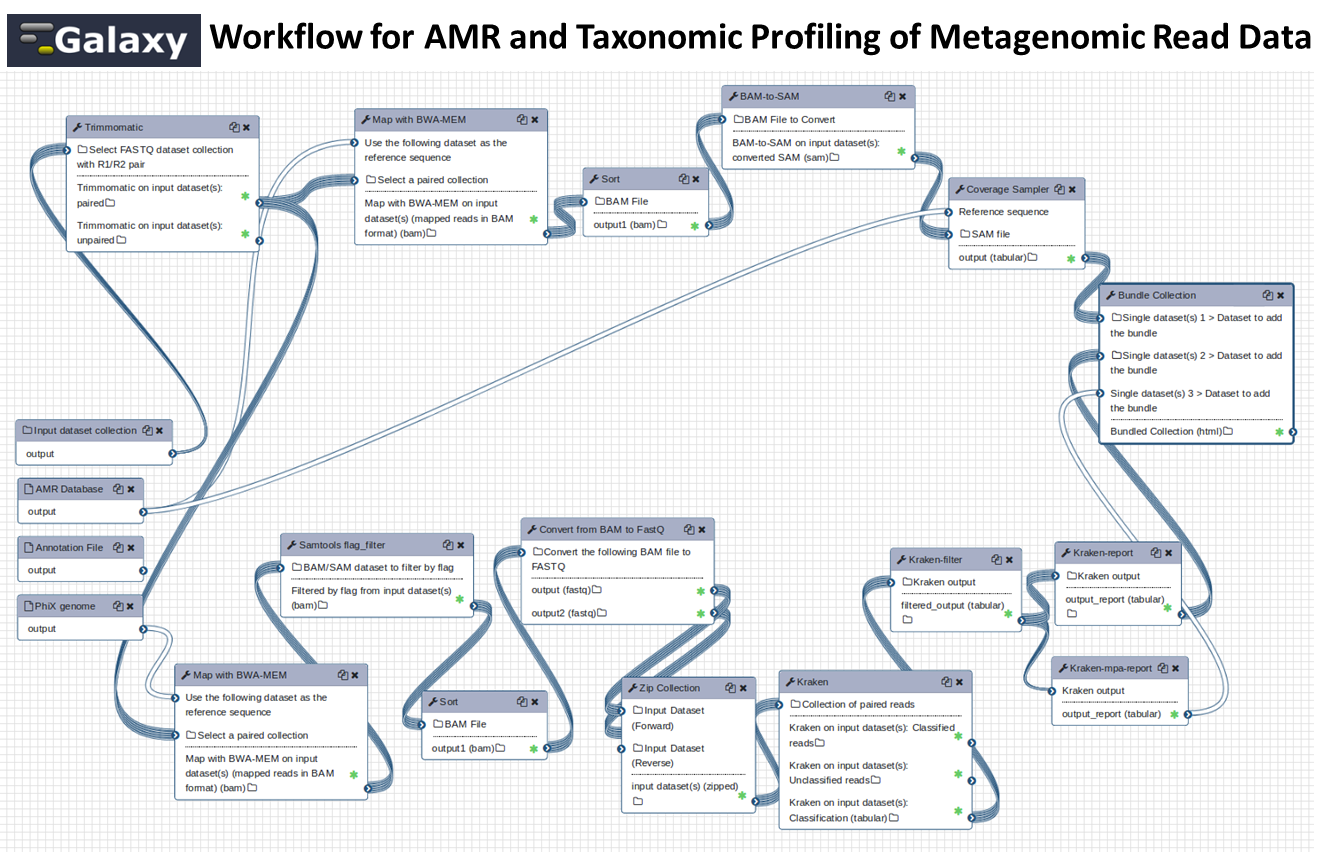
**

**Figure S2** **Galaxy workflow (**[**https://galaxyproject.org/**](https://galaxyproject.org/)**) for antimicrobial resistance (AMR) and taxonomic profiling of metagenomics sequencing read data.** Input data includes collection of forward and reverse sequence reads (fastq), AMR gene data base (fasta), AMR gene annotations (csv), and PhiX174 genome (fasta). Following read trimming and quality filtering via Trimmomatic data is channeled into two parallel work flows for taxonomic and resistome profiling using a series of tools indicated in the figure (grey headings). The resulting data files are downloaded (as a bundle collection) for further analyses.

**Tools, databases and versions**:

- Trimmomatic version 0.36
- The Kraken taxonomic classification tools (version 0.10.5 beta)
- BWA-MEM version 0.7.17.1
- Coverage sampler/Resistome analyzer <https://megares.meglab.org/amrplusplus/latest/html/>
- Samtools <http://www.htslib.org/> (git clone git://github.com/samtools/samtools.git)
- MEGARes database version 1.01
- phage phiX174 (GenBank accession NC_001422.1)
